# Comparative analysis of European bat lyssavirus 1 pathogenicity in the mouse model

**DOI:** 10.1371/journal.pntd.0005668

**Published:** 2017-06-19

**Authors:** Elisa Eggerbauer, Florian Pfaff, Stefan Finke, Dirk Höper, Martin Beer, Thomas C. Mettenleiter, Tobias Nolden, Jens-Peter Teifke, Thomas Müller, Conrad M. Freuling

**Affiliations:** 1 Institute of Molecular Virology and Cell Biology, Friedrich-Loeffler-Institut, Federal Research Institute for Animal Health, Greifswald-Insel Riems, Germany; 2 Institute of Diagnostic Virology, Friedrich-Loeffler-Institut, Federal Research Institute for Animal Health, Greifswald-Insel Riems, Germany; 3 Department of Experimental Animal Facilities and Biorisk Management, Friedrich-Loeffler-Institut, Federal Research Institute for Animal Health, Greifswald-Insel Riems, Germany; University of Texas Medical Branch, UNITED STATES

## Abstract

European bat lyssavirus 1 is responsible for most bat rabies cases in Europe. Although EBLV-1 isolates display a high degree of sequence identity, different sublineages exist. In individual isolates various insertions and deletions have been identified, with unknown impact on viral replication and pathogenicity. In order to assess whether different genetic features of EBLV-1 isolates correlate with phenotypic changes, different EBLV-1 variants were compared for pathogenicity in the mouse model. Groups of three mice were infected intracranially (i.c.) with 10^2^ TCID50/ml and groups of six mice were infected intramuscularly (i.m.) with 10^5^ TCID50/ml and 10^2^ TCID50/ml as well as intranasally (i.n.) with 10^2^ TCID50/ml. Significant differences in survival following i.m. inoculation with low doses as well as i.n. inoculation were observed. Also, striking variations in incubation periods following i.c. inoculation and i.m. inoculation with high doses were seen. Hereby, the clinical picture differed between general symptoms, spasms and aggressiveness depending on the inoculation route. Immunohistochemistry of mouse brains showed that the virus distribution in the brain depended on the inoculation route. In conclusion, different EBLV-1 isolates differ in pathogenicity indicating variation which is not reflected in studies of single isolates.

## Author summary

European bat lyssavirus 1 (EBLV-1) is one of fourteen officially recognized lyssavirus species causing rabies, a zoonosis resulting inevitably in death once clinical signs appear. EBLV-1 is responsible for most bat rabies cases detected in Europe, and spill-over infections in humans highlight its zoonotic potential. In our study, we compared eight genetically diverse EBLV-1 isolates in the mouse model using various routes of inoculation. Although EBLV-1 isolates displayed very high sequence conservation, significant differences in pathogenicity, i.e. in incubation periods and mouse survival, were observed. Furthermore, depending on the inoculation route the clinical picture as well as the virus antigen distribution within the brain varied. Thus, transfer of results obtained with single isolates to the whole lyssavirus species can be misleading, and results indicating reduced pathogenicity obtained with single EBLV-1 isolates in previous studies have to be carefully interpreted.

## Introduction

Rabies is an acute, progressive and incurable viral encephalitis, caused by negative strand RNA viruses of the Lyssavirus genus belonging to the order Mononegavirales, family Rhabdoviridae, which is transmitted by bites of infected mammals. Taxonomically, the etiological agents are classified into 14 officially recognized and two yet unassigned lyssavirus species [1–3]. Intriguingly, for the great majority of lyssaviruses bats (*Chiroptera*) are the reservoir leading to the assumption that bats are the true ancestral host of all lyssaviruses [4]. Hence, rabies is the most significant viral zoonosis associated with bats as almost all bat lyssaviruses have caused fatal spillovers into humans and terrestrial mammals [5–7].

Bat rabies in Europe was initially discovered in 1954 [8]. Subsequent virus characterization using monoclonal antibodies showed that the viruses isolated from bats at the time were distinct from classical rabies virus (RABV) and were assigned as European bat lyssaviruses types 1 and 2 (EBLV-1 and -2) [9, 10]. In recent years, novel lyssavirus species have been detected in European bats, namely West Caucasian bat lyssavirus (WCBV) [11], Bokeloh bat lyssavirus (BBLV) [12] and Lleida bat lyssavirus (LLBV, [3]).

European bat lyssavirus 1 (EBLV-1) is the most common of the five lyssavirus species circulating in European bats and responsible for the majority of all recorded bat rabies cases in Europe [13]. The reservoir hosts of EBLV-1 are the serotine bat (*Eptesicus serotinus*) and the Isabelline serotine bat (*Eptesicus isabellinus*) [14], but occasional cases were also found in other bat species, e.g. the brown long-eared bat (*Plecotus auritus*), the common pipistrelle (*Pipistrellus pipistrellus*), and the Nathusius' pipistrelle (*Pipistrellus nathusii*), [15] as well as in sheep [16], cats [17] and a stone marten [18]. The zoonotic potential of EBLV-1 is demonstrated by the fact that at least two confirmed human cases occurred in Russia and the Ukraine [19, 20].

The nucleoprotein (N)-gene is the most conserved gene across all lyssaviruses [21] and frequently used for phylogenic analyses since its diversity allows a good separation between lyssavirus species [22]. Based on partial N-gene sequences, EBLV-1 can be divided into two distinct sublineages EBLV-1a and EBLV-1b, the first predominantly found in Central and Eastern Europe, the second in southwest Europe [23, 24]. Recently, a third sublineage of EBLV-1 comprising isolates from the Isabelline bat on the Iberian peninsula has been proposed [14]. Genetically, EBLV-1 isolates show a very high nucleotide identity above 99% in EBLV-1a and 98% in EBLV-1b, respectively. The overall heterogeneity at nucleotide level is less than 3.3% [23].

Thus far, full genome sequences have never been assessed for genomic differences although evidence for those was found in the form of insertions and deletions (indels) in areas usually not sequenced for phylogenetic analysis. For example, a six nucleotide insertion was identified in the 3′untranslated region (UTR) of EBLV-1b isolates [25, 26] and a single nucleotide insertion in EBLV-1a isolates in the same area. Furthermore, a 35 nucleotide deletion was found in the G-L intergenic region of one EBLV-1a isolate [26]. The potential impact of these genomic differences as well as the influence of the overall genetic diversity within EBLV-1 sublineages on the pathogenicity remains elusive, since so far only single representative EBLV-1 isolates were used in pathogenicity studies. Those were usually comparative studies of different lyssavirus species or studies aimed at efficacy testing of antibodies and vaccines [27–33]. However, differences in pathogenicity within other lyssavirus species had been observed, i.e. for Lagos bat virus (LBV) and Rabies virus (RABV) [34–36]. Against this background and the conundrum of reduced pathogenicity in experimental animal studies on one side and human casualties on the other, this study aimed at analyzing different EBLV-1 isolates representing all three sublineages to assess variability in pathogenicity.

## Materials and methods

### Ethics statement

All in vivo work was performed according to European guidelines on animal welfare and care according to the Federation of European Laboratory Animal Science Associations (FELASA). The characterization of lyssaviruses in the mouse model was reviewed and approved by the review board of the Landesamt für Landwirtschaft, Lebensmittelsicherheit und Fischerei M-V (LALLF, Document-ID: AZ LALLF.M-V/TSD/7221.3–2.1-002/11).

### Viruses

Ten lyssavirus isolates originating from the archive of the Friedrich-Loeffler-Institute (FLI) Riems were included in the study. Two viruses belonged to RABV and eight isolates to EBLV-1, consisting of five EBLV-1a isolates, two EBLV-1b isolates and a single EBLV-1 isolate of the proposed third sublineage, here termed EBLV-1c. Five isolates have already been described previously including EBLV-1 isolates with insertions and deletions [25, 26], a distant EBLV-1a isolate and an EBLV-1a isolate responsible for a human rabies case in Russia [19]. Properties of selected isolates are detailed in Table 1. Cell lines used in this study for viral propagation, titration, replication kinetics and serology were obtained from the Collection of Cell Lines in Veterinary Medicine (CCLV) established at FLI, Riems, Germany.

**Table 1:** Isolates and viruses used in the study including details of their respective characteristics, year of isolation, host and origin.

Lab ID	Name	Viral species	Characteristics	Year	Host	Origin	Accession numbers
13454	13454_EBLV-1a_ref	EBLV-1a	Isolate used for the infection of foxes and ferrets [27, 33] available as a recombinant virus [37],	2000	*Eptesicus serotinus*	Germany	LT839615
5782	5782_EBLV-1a_del	EBLV-1a	35nt deletion in G-L region [26]	2001	unknown	Germany	LT839611
5776	5776_EBLV-1a_ins	EBLV-1a	1nt (A) insertion in N-P region [26]	2001	unknown	Germany	LT839614
976	976_EBLV-1a_dist	EBLV-1a		1992	*Pipistrellus nathusii*	Germany	LT839610
13027	13027_EBLV-1a_Yuli	EBLV-1a	human rabies case (Yuli)[19]	1982	human	Russia	LT839613
20174	20174_EBLV-1b	EBLV-1b	-	2008	*Eptesicus serotinus*	Germany	LT839609
5006	5006_EBLV-1b_ins	EBLV-1b	6nt (AAAAGA) insertion in N-P region, as described before [25]	2000	*Eptesicus serotinus*	Germany	LT839612
13424	13424_EBLV-1c	EBLV-1c	-	1989	unknown	Spain	LT839608
35009	35009_RABV_CVS	RABV	fixed RABV strain, challenge virus standard (CVS), batch 1, ANSES Nancy, France	1996	-	-	LT839616
5989	5989_RABV_dog_azerb	RABV	RABV field strain, used in experimental studies [38, 39]	2002	dog	Azerbaijan	LN879480

A, Adenine;

G, Guanine;

Brain samples of mice inoculated with isolates highlighted in grey were subjected to IHC.

### Viral propagation and replication kinetics

Prior to mouse inoculation virus stocks were produced for all lyssaviruses. Viral propagation, titration and replication kinetics of the lyssavirus isolates were conducted on mouse neuroblastoma cells (Na 42/13, CCLV-RIE 0229). For virus propagation cells were infected at a multiplicity of infection (MOI) of 0.001, incubated at 37°C and 5% CO_2_ for at least for 72 hours. When 100% of the monolayer was infected supernatant virus was harvested. Depending on the isolates an additional passaging was required. After harvesting infectious virus titres were determined by endpoint titration, calculated using the Spearman-Karber method [40] and expressed as tissue culture infective dose 50 (TCID50).

Replication kinetics were determined by one step and two step growth curves. For each isolate Na 42/13 cells were infected at MOIs of 0.01 and 3, and subsequently incubated at 37°C and 5% CO2 for 96 hours. Supernatant virus titres were determined at 0, 16, 24, 48, 72 and 96 hours post infection by endpoint titration. For each isolate two biological as well as two technical replicates were done.

### Mouse inoculation and sampling

Three to four week old female Balb/c mice (Charles River, Germany) were inoculated with the selected isolates (Table 1) using three different inoculation routes and two different viral doses. While groups for intramuscular (i.m.) and intranasal (i.n.) inoculation consisted of six animals, three animals were used in positive (intracranial, i.c.) as well as in negative (mock infected) control groups. Groups were housed in individual cages and mice had access to water and food ad libitum.

For each isolate two groups of mice were inoculated i.m. into the right or left gluteal muscle using high (10^5^ TCID50/30μl) and low (10^2^ TCID50/30μl) viral doses. Because viral propagations of isolates 5989_RABV_dog_azerb and 20174_EBLV-1b did not yield viral titres of 10^5^ TCID50/30μl, undiluted supernatant with titres of 10^4^ TCID50/30μl for both isolates were used as a high dose for i.m. inoculation. Additionally, one group of mice was i.n. inoculated with 5 μl of viral suspensions (10^2^ TCID50/10μl) in each nostril using a pipette. Positive and negative controls were inoculated i.c. either using 10^2^ TCID50/30μl of viral suspension or 30μl of cell culture medium.

All mice were marked with earclips for identification and monitored daily for 45 days post infection (dpi). Weight and clinical scores, ranging from zero up to four, were recorded daily (see S1 Table). With onset of clinical signs mice were examined twice daily. At a clinical score of three or when the weight loss exceeded 20% mice were anaesthetized using Isoflurane and euthanized through cervical dislocation. All remaining animals were euthanized 45 days after inoculation.

Upon euthanasia, brain samples were taken from all mice. From animals inoculated with six representative isolates (Table 1) that died during the observation period half of the brain was fixed in 4% paraformaldehyde (PFA) for additional immune-histochemical analysis. Furthermore, blood was collected by heart puncture in 600μl tubes (BD Microtainer, SST Tubes), allowed to settle for at least 30 min and centrifuged for 3 min at 10,000×g. Afterwards, the serum was transferred to 1.5 ml tubes and stored at -70°C until serological testing.

### Detection of viral antigen

Brain samples were tested for the presence of lyssaviral antigen using the fluorescence antibody test (FAT) as described elsewhere [41]. In brief, brain smears were heat-fixed on slides followed by staining with a FITC-conjugated polyclonal antibody (SIFIN, Berlin, Germany) for 30 minutes. Slides were examined under a fluorescence microscope and considered positive if green fluorescence was present. Defined positive and negative controls were included in every test run.

Furthermore, paraffin embedded brain samples of selected animals (see above, Table 1) were subject to histochemical analysis as described before [42, 43]. Briefly, after fixation in 4% PFA and embedding in paraffin wax (FFPE), samples were cut in 3μm thick paramedian sections and dewaxed, followed by immunohistochemistry (IHC) using an anti-nucleoprotein (N) polyclonal rabbit serum N161-5 [37]. The amount of viral antigen in the complete paramedian cross sections as well as in different brain regions i.e. the medulla, the cerebellum, the cortex and the olfactory bulb was semi-quantitatively analyzed using a four plus scoring system.

### Serological assays

Sera were tested for the presence of virus neutralizing antibodies (VNAs) in a modified rapid fluorescence focus inhibition test (RFFIT) [44, 45] using a homologous RABV and EBLV-1 isolate as test virus and BHK21-BSR/5 (CCLV-RIE 0194/260) and BHK21-C13 (CCLV-RIE 017971113) cells, respectively. The WHO international standard immunoglobulin (2nd human rabies immunoglobulin preparation, National Institute for Standards and Control, Potters Bar, UK) adjusted to 0.5 and 1.5 international units (IU) for RABV and EBLV-1, respectively, was used as positive control [45]. A naive bovine serum was used as negative control. The potential immune response to infection was assessed qualitatively and sera were considered positive if neutralizing activity was equal or above the respective positive controls.

### Statistical analysis

Statistical analyses were performed using GraphPad Prism version 7.00 (GraphPad Software, La Jolla California USA) with p-values < 0.05 considered significant. Replication kinetics were analyzed by calculating the area under the curve (AUC) followed by statistical analysis using an ordinary one-way ANOVA combined with Tukey′s multiple comparison test. To infer statistical differences in survival rates the Mantel-Cox test (log-rank test) was used, while incubation periods were evaluated using the same statistical analysis as for the replication kinetics. For statistical analyses of results obtained in IHC data were stratified in respect to (i) inoculation route and (ii) the different isolates following i.m. inoculation. To this end, the Kruskal-Wallis test was applied and adjusted p-values for direct comparison of two groups were obtained using Dunn′s multiple comparison test.

### Full genome sequencing

Total RNA was extracted from 2 ml cell culture supernatant using TriFast (VWR Peqlab, Erlangen, Germany) together with the RNeasy Mini Kit (Qiagen, Hilden, Germany) and DNase (Qiagen) treatment as recommended by the supplier. The RNA was further concentrated using Agencourt RNAclean XP beads (Beckman Coulter) and used as input for the preparation of cDNA sequencing libraries as described elsewhere [46]. Sequencing was carried out on an Illumina MiSeq instrument using the MiSeq reagent kit, version 3 (Illumina, San Diego, USA) in 2x300 bp paired end mode. A combination of reference based mapping along appropriate references and de-novo assembly as implemented in the 454 software suite (version 3.0, Roche) was used to generate EBLV-1 and CVS full-genomes. These sequences were annotated in Geneious [47], version 10, http://www.geneious.com] and submitted to the European Nucleotide Archive under study number PRJEB20390 (Table 1). For sequence comparison and phylogenetic analysis, 7 full-length EBLV-1 reference sequences were aligned with sequences obtained in this study for a total number of 15 sequences, using the MAFFT plugin in Geneious. A maximum-likelihood tree was calculated from this alignment using the optimal substitution model GTR+G and 1000 bootstrap replicates as incorporated in MEGA7 [48]. The protein coding regions were translated in amino acid sequences and screened for amino acid exchanges in known pathogenicity determining sites.

## Results

### Replication kinetics

All viruses grew to maximum titres ranging between 10^6.5^ and 10^9^ TCID50/ml with the highest titres at different time points observed for 35009_RABV_CVS for MOI 0.01 while 5006_EBLV-1b_ins had the lowest titres for MOI 3 (S1 Fig). However, the differences observed were below the level of significance.

### Incubation periods

**Fig 1:**
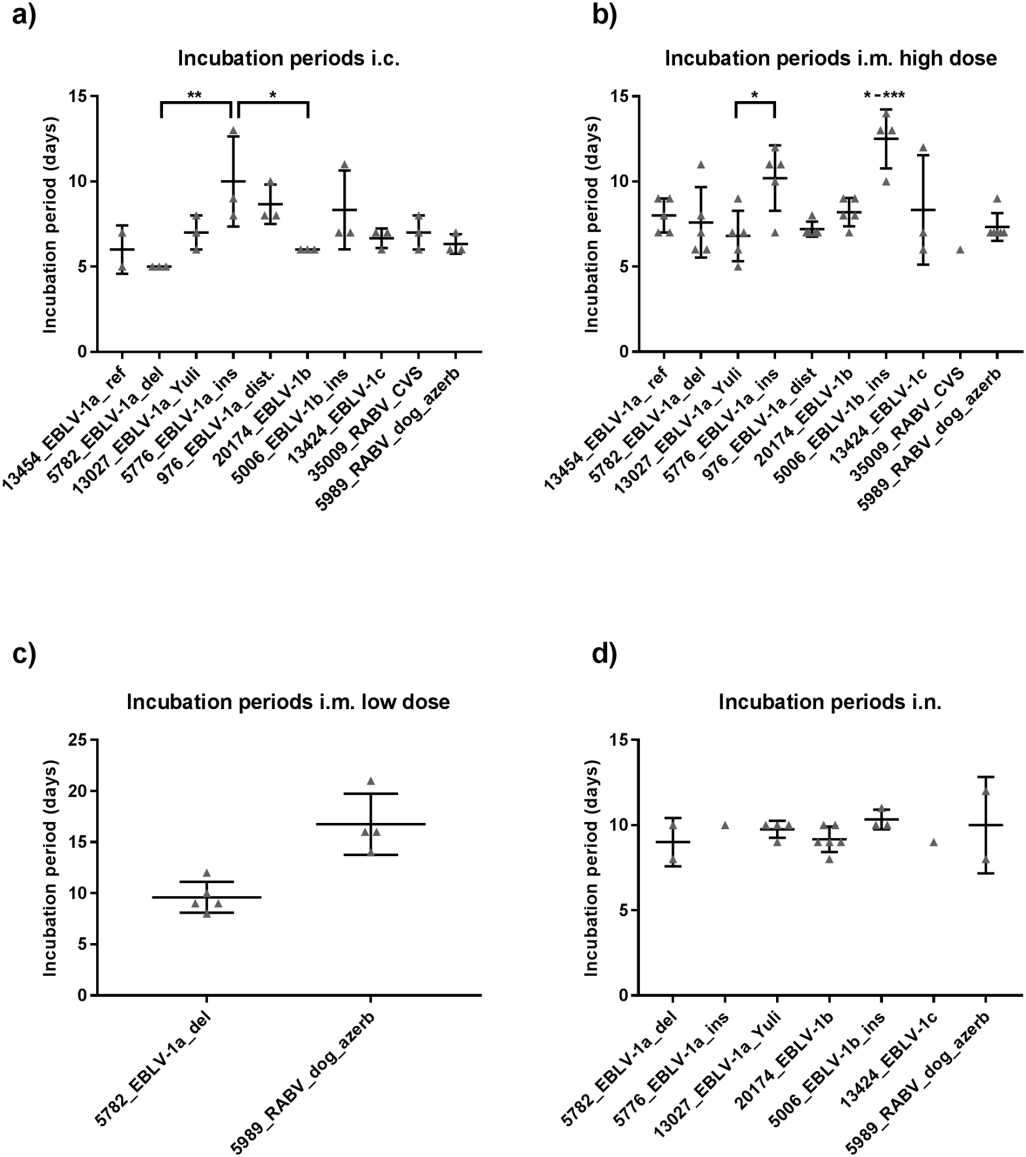
Incubation periods following i.c., i.m., and i.n. inoculation. Individual incubation periods of mice following a) i.c. inoculation, b) i.m. inoculation with high doses, c) i.m. inoculation with low doses and d) i.n. inoculation of the isolates (grey triangles) with n = 3 for i.c. and n = 6 for i.m. and i.n. inoculated mice. Mean and standard deviation (SD) of the isolates are indicated by the horizontal bars and whiskers, respectively. P-values, obtained using an ordinary one-way ANOVA combined with Tukey′s multiple comparison test, are indicated by asterisks, with * indicating p-values ≤ 0.05, ** ≤ 0.01 and *** ≤ 0.001.

After i.c. inoculation first clinical signs within the groups appeared between five and eight dpi, while the mean incubation periods varied between five and ten days. Significant differences were observed between isolate 5776_EBLV-1a_ins with a mean incubation period 10 dpi and isolates 5782_EBLV-1a_del and 20174_EBLV-1b with mean incubation periods of 5 and 6 days respectively (p-values: 0.0067 & 0.0439) (Fig 1a).

Groups of mice inoculated i.m. with high doses of the lyssavirus isolates started to show clinical sings between 5 and 10 dpi. Mean incubation periods varied between the groups from 6 to 13 dpi with significant differences between EBLV-1 isolates (p-value: 0.0003). 5006_EBLV-1b_ins had significant longer incubation periods compared to all other EBLV-1 isolates (p-values: 0.0004–0.0433) with the exception of 5776_EBLV-1a_(ins) (p-value: 0.4443). For the latter isolate, a significant difference in the mean incubation period could be observed compared to 13027_EBLV-1a_Yuli (p-value: 0.0483, Fig 1b). Following i.m. inoculation with low doses only mice inoculated with isolates 5989_RABV_dog_azerb and 5782_EBLV-1a_del developed clinical signs after an average of 17 and 9 dpi, respectively (Fig 1c). Mean incubation periods after i.n. inoculation ranged between 8 and 10 dpi (p-value >0.05, Fig 1d).

### Clinical sings

The clinical picture of mice inoculated i.c. usually included general signs like weight loss, ruffled fur, a hunched back and slowed movements. In rare occasions spasms, aggressiveness or increased activity was observed.

Following i.m. inoculation the clinical signs usually started in the inoculated limb with either spasms in mice inoculated with EBLV-1 resulting in hypermetria or a wobbly gait, or paralysis in mice inoculated with RABV and eventually included the second hind limb. At this point, ruffled fur, a hunched back or trembling were commonly observed in RABV infected animals, but only sporadically in mice inoculated with EBLV-1. Aggressive behavior was rarely observed and restricted to 5006_EBLV-1b_ins and 976_EBLV-1a_dist infected mice.

Intranasal inoculation of EBLV-1 isolates resulted in clinical signs like tameness, aggressiveness, circular movement and occasionally automutilation. Notably, of the two mice which developed clinical signs following i.n. inoculation with 5989_RABV_dog-azerb one developed tremor whereas the other did not show any clinical signs, except for progressive weight loss, eventually leading to euthanasia.

### Pathogenicity and survival

All mice inoculated i.c. with the isolates developed clinical signs and died, except for one mouse inoculated with 13454_EBLV-1a_ref (S2 Fig).

**Fig 2:**
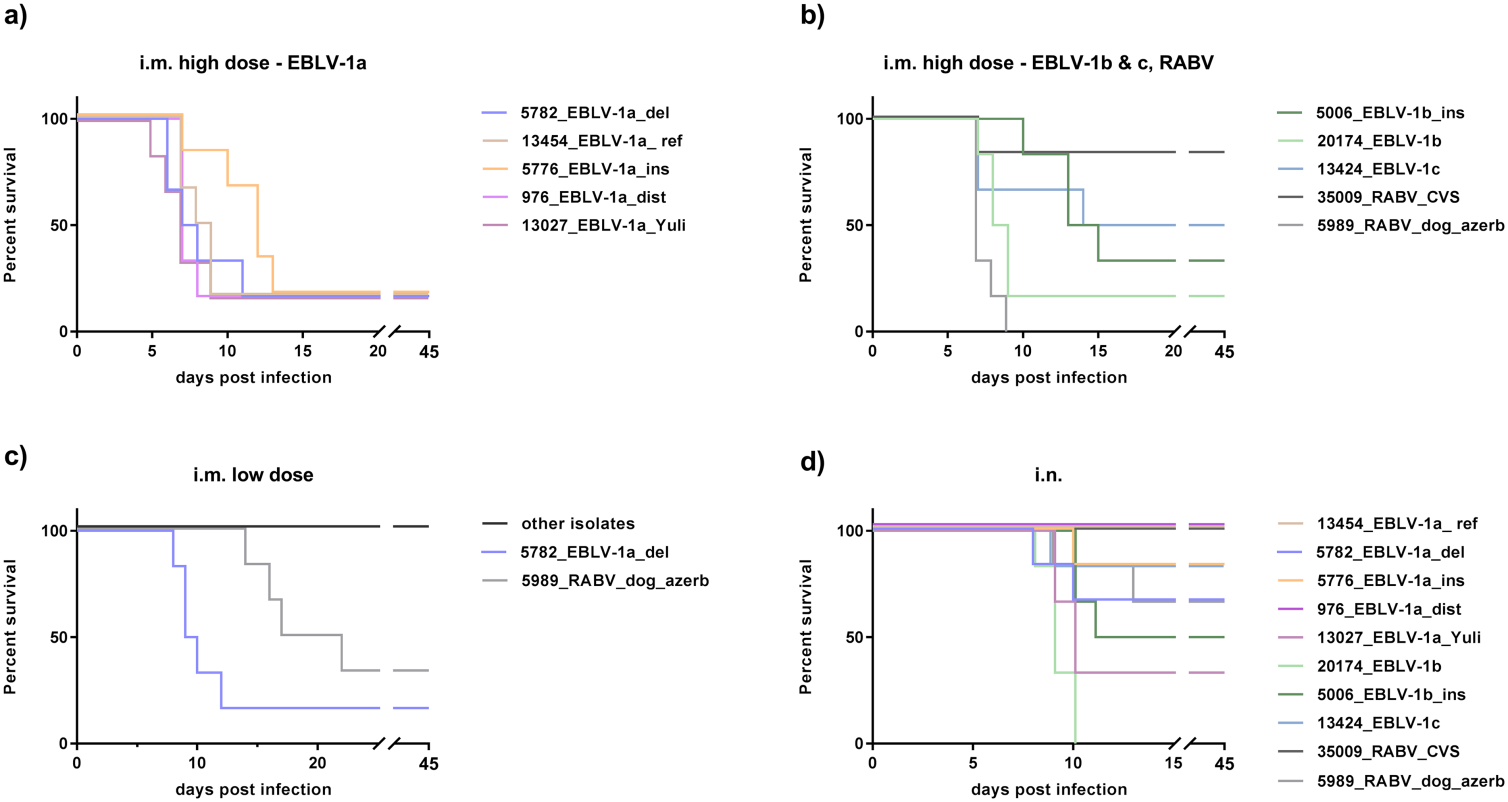
Kaplan-Meyer survival plots following i.m. infection with high doses (a, b), low doses (c) and i.n. infection (d). Six Balb/c mice were inoculated per group.

Survival among i.m. high dose infected EBLV-1 groups varied between 50% for 13424_EBLV-1c and 17% for all other EBLV-1 isolates except 5006_EBLV-1b_ins (33%) (p-values > 0.05). After RABV infection 83% (35009_RABV_CVS) and 0% (5989_RABV_dog azerb) of mice survived, respectively (p-value: 0.004). Only isolates 5989_RABV_dog_azerb and 5782_EBLV-1a_del were pathogenic following i.m. inoculation with a low dose, resulting in a significant difference in survival between the RABV isolates (p-value: 0.02) as well as between 5782_EBLV-1a_del and the other EBLV-1 isolates (p-value < 0.0001, Fig 2a–2c).

I.n. inoculation resulted in significant differences in survival within the EBLV-1a isolates and within the EBLV-1b + 1c isolates (p-value < 0.03). Compared to isolates 13454_EBLV-1a_ref and 976_EBLV-1a_dist, isolate 13027_EBLV-1a_Yuli displayed a significant lower survival (p-value: 0.0191). No mice survived following inoculation with isolate 20174_EBLV-1b which resulted in a significant difference in survival compared to isolates 5006_EBLV-1b_ins and 13424_EBLV-1c (p-value: 0.0055 & 0.0061) as well as compared to isolate 13454_EBLV-1a_ref, 976_EBLV-1a_dist (p-values: 0.0008) and isolates 5776_EBLV-1a_ins and 5782_EBLV-1a_del (p-values: 0.0024 & 0.028). Survival following inoculation with the RABV isolates was similar in both groups (p-value>0.05) (Fig 2d)

All mock infected mice did not show clinical sings and survived until the end of the observation period.

### Antigen detection

**Fig 3:**
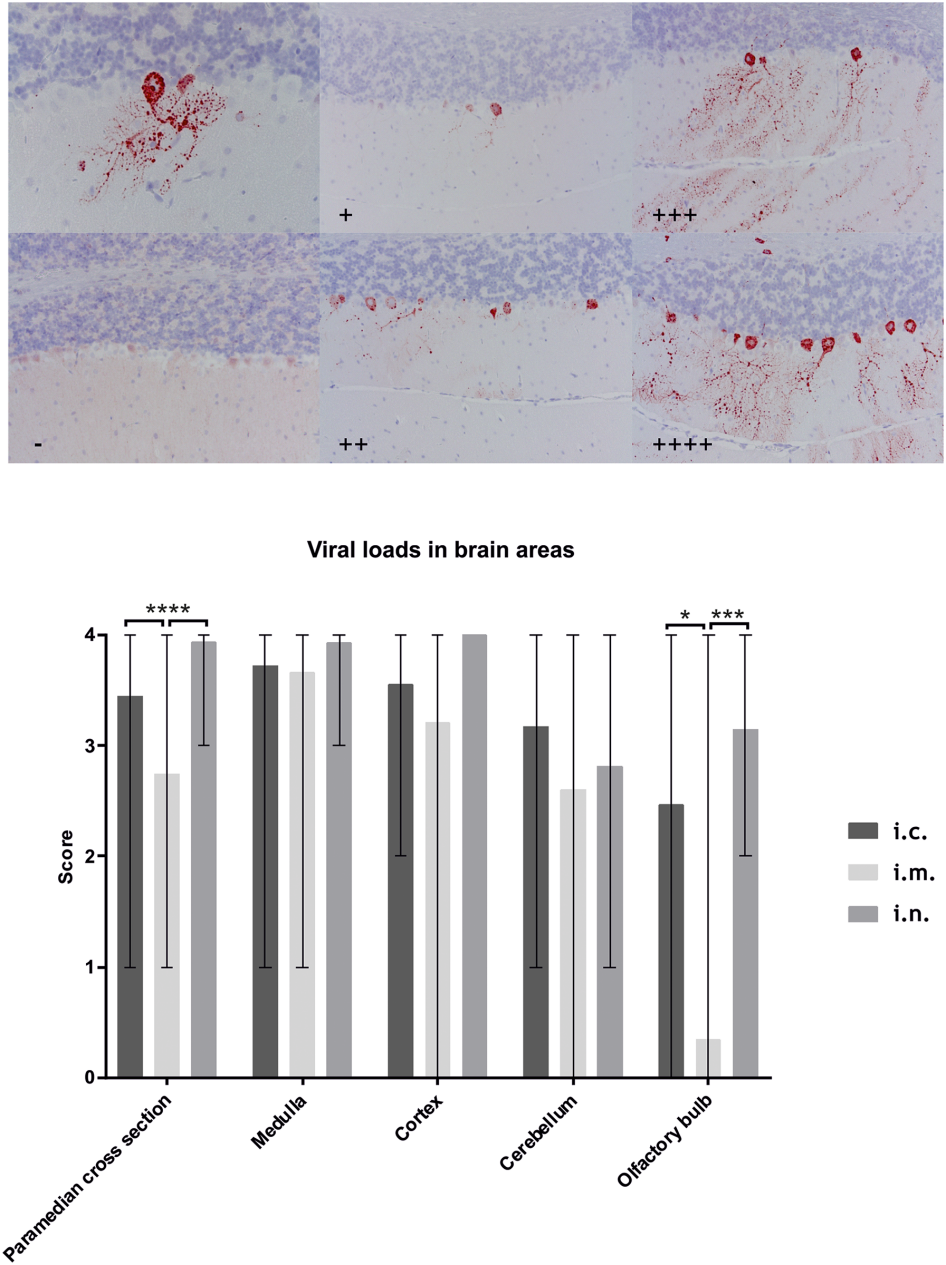
IHC score for the amount of lyssavirus antigen and antigen distribution in the different brain regions. a) IHC pictures of the cerebellum depicting a lyssavirus antigen positive Purkinje cell (upper right) and the score ranging from negative (-) up to four plus (++++). b) Distribution of viral antigen in the paramedian cross sections, the medulla, the cortex, the cerebellum and the olfactory bulb with respect to the inoculation route. Whiskers indicate the range of the data sets. P-values below 0.5 are indicated by asterisk, * indicating p-values ≤ 0.05, ** ≤ 0.01 and *** ≤ 0.001.

All mice which were euthanized or died during the experimental stage were positive while all animals that were killed at the end of the observation period were negative using FAT. With IHC, the amount of antigen in the brain varied depending on the inoculation route, with a lower antigen content in the paramedian cross sections and the olfactory bulb following i.m. inoculation compared to i.c. (p-values: 0.0379 & <0.0001) and i.n. inoculation (p-values: 0.0003 & <0.0001; Fig 3a and 3b). No significant difference could be observed upon comparison of the isolates following intramuscular inoculation (p-values: >0.58).

### Serology

VNAs were detected both in survivors as well as in animals that succumbed to infection after i.c and i.m inoculation. Following i.m. high dose inoculation seroconversion of the mice varied between 17% and 100%. In groups inoculated with isolates 5006_EBLV-1b_ins and 35009_RABV_CVS all mice which succumbed to disease did not seroconvert whereas all survivors did. Overall seroconversion was higher following i.m. inoculation with high doses (58% for EBLV-1 and 92% for RABV) compared to low doses (19% for EBLV-1 and 33% for RABV) (S3 Fig). Following 5989_RABV_dog_azerb i.m. low dose infection only mice which succumbed to disease seroconverted whereas the opposite was true for isolate 5782_EBLV-1a_del, where only survivors seroconverted. None of the animals inoculated i.n. as well as the mock infected control group developed VNAs.

### Comparison of nucleotide and amino acid sequences

**Fig 4:**
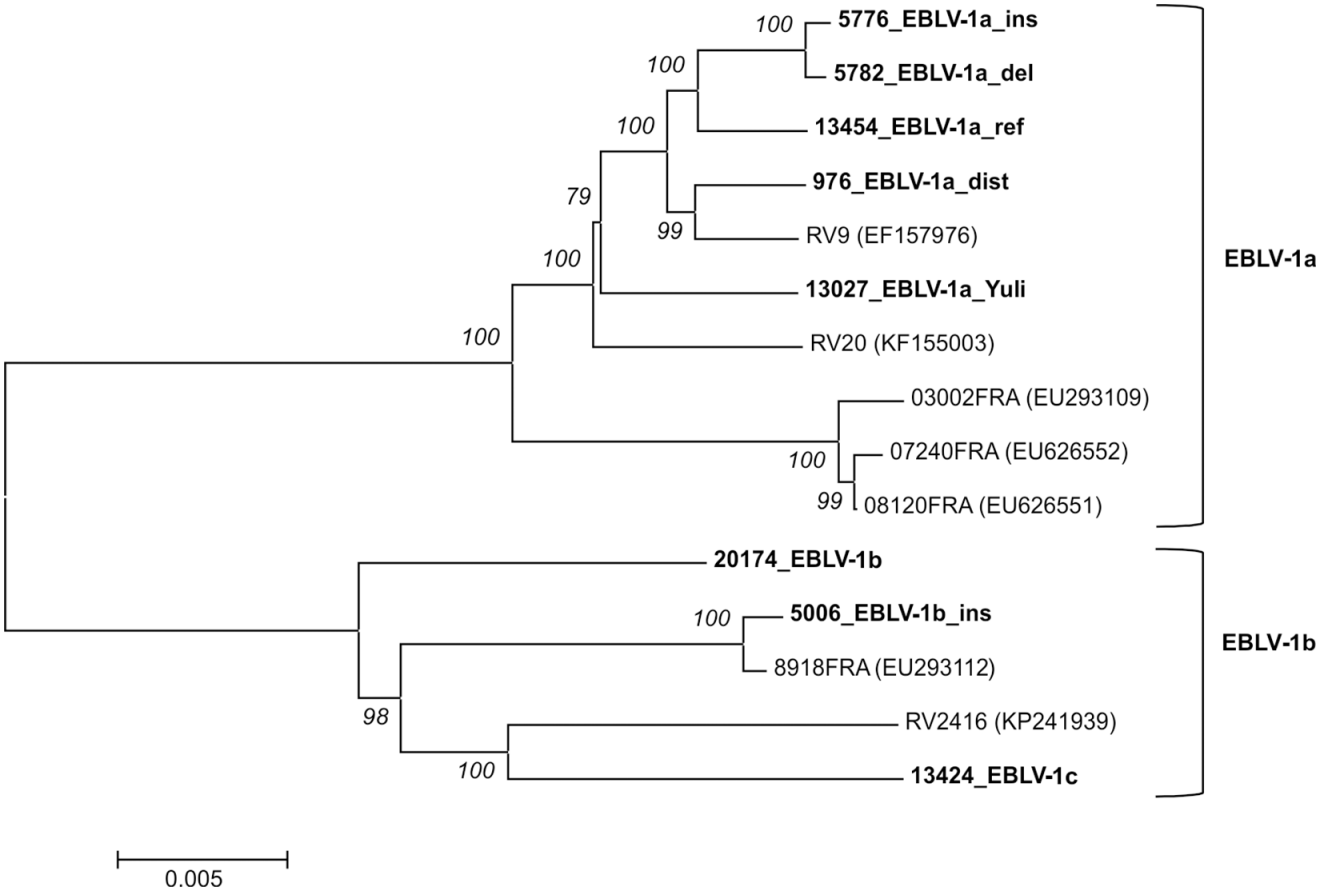
Phylogenetic relationship of EBLV-1 isolates inferred from all currently available full-length genome sequences. The observed phylogenetic grouping is in accordance with the classification into the distinct sublineages EBLV-1a and EBLV-1b. For EBLV-1c currently only one full-length genome sequence is available. Sequences obtained in this study are highlighted in bold type and bootstrap support values are indicated in italics.

Sequence analysis of the EBLV-1 full genome sequences revealed nucleotide identities within the lineages above 98.8% for EBLV-1a and above 97.4% for both EBLV-1b and EBLV-1c. Also, the heterogeneity between the groups was below 5%, as visualized in the branching pattern of the phylogenetic tree (Fig 4). For isolate 5006_EBLV-1b_ins an additional single nucleotide insertion (nt) in the G-gene UTR (position 3308) was discovered. In total the number of single nucleotide polymorphisms was 567. At amino acid (aa) level a total of 71 aa exchanges were found among the EBLV-1 isolates. Of those, 28 resulted in a change of the respective aa property (S2 Table). Differences in the aa sequences of the EBLV-1 isolates could be observed in two known pathogenicity determining sites (S3 Table). One aa exchange was in the phospoprotein of isolate 5006_EBLV-1b_ins at position 176 where Serine was exchanged with Proline (S176P). Furthermore in the glycoprotein at position 503 of the EBLV-1a isolates a Glycine was present, whereas the EBLV-1b and 1c isolate had a Serine at this position. All isolates had a glycosylation site in the glycoprotein at position 319, while both RABV isolates had additional sites at position 37 and isolate 35009_RABV_CVS at position 204.

## Discussion

Pathogenicity studies are essential e.g. to characterize individual viruses and to understand virus-host interactions. The latter studies are preferentially performed in the respective reservoir host. Unfortunately, most lyssaviruses including EBLV-1 have their reservoir in bats, with evident challenges in performing studies in those bat species. Although initial studies were performed with EBLV-2 in Daubenton’s bats [49] and with EBLV-1 in the Serotine bat [50], the protected status of these animals, as well as their challenging husbandry and handling precludes using these species for comparative analyses. As an alternative, infection of mice was established as a model to study lyssavirus pathogenesis.

Most pathogenicity studies were performed using RABV [51], demonstrating differences in virus characteristics depending on the isolates used [35, 36, 51]. Comparative analyses of different lyssavirus isolates within one species were also published for LBV, where distinct differences in pathogenicity between isolates were also recorded [34]. Although some studies included EBLV-1, only single isolates were used as representatives. In those studies, various mouse breeds, application routes, cells for virus propagation and viral doses were used. Furthermore, titres were usually expressed as MICLD50/MLD50 (S4 Table), thus preventing direct comparison. Therefore, in this study the pathogenicity of eight EBLV-1 isolates was compared under identical conditions. All known sublineages were included as well as isolates containing insertions and deletions attempting to represent the diversity of this lyssavirus species.

We observed significant differences in the pathogenicity between the EBLV-1 isolates, with isolate 5782_EBLV-1_del displaying a higher pathogenicity following i.m. inoculation with a low dose compared to all other EBLV-1 isolates. This is remarkable, considering that the nucleotide sequence is 99.6% identical with isolate 5776_EBLV-1a_ins which was not pathogenic after i.m. low dose application. Overall, there is a high nucleotide identity among the EBLV-1 isolates and the only distinctive feature of 5782_EBLV-1a_del on nucleotide level is the 35nt deletion in the pseudogene region as described before [26]. On protein level no aa exchange in isolate 5782_EBLV-1a_del could be observed which would explain this increase in pathogenicity compared to the other EBLV-1 isolates (S3 and S4 Tables).

While deletions or insertions in the pseudogene region of fixed RABV strains did not change their pathogenicity after intracranial inoculation [52, 53], experimental studies using chimeric viruses revealed that the pseudogene contributes to neuroinvasiveness after peripheral infection [54]. Another possible reason for this difference could be an aa exchange in a so far unknown pathogenicity determining site of this EBLV-1 isolate. In order to identify and verify responsible differences further studies using reverse-genetics are warranted.

Following i.n. inoculation, interestingly, the majority of EBLV-1 isolates displayed a higher pathogenicity compared to i.m. low-dose inoculation, although the same viral dose was used. Within i.n. inoculated mice significant differences were observed, with survival rates of the isolates varying between 0% and 100% (Fig 2d). However, there was no correlation with the pathogenicity following i.m. inoculation, as here most EBLV-1 isolates were similar pathogenic. Isolate 5782_EBLV-1a_del which was highly pathogenic following i.m. inoculation with a low dose, was less pathogenic following i.n. inoculation compared to isolate 20174_EBLV-1b. Even isolates 20174_EBLV-1b and 5006_EBLV-1b_ins with a high identity on nucleotide level of 97.8% had a difference in mortality of 50%. It is unclear why certain viruses are pathogenic via i.n. inoculation while others seem to be apathogenic.

Previous studies investigating intranasal or aerosol infection used the fixed RABV strain CVS and EBLV-2 with different results regarding pathogenicity [55–58]. But although these results seemed to sometimes contradict each other, it has to be noted that the studies were all designed differently. In fact, one study showed that the pathogenicity following i.n. infection depends on a variety of factors, for example the amount and exact administration of the inoculum [59]. In our study the pathogenicity of different isolates of the same lyssavirus species were investigated under the same conditions and differences in pathogenicity were still observed. Intranasal inoculation is an artificial infection route, even though it has been proposed for transmission of lyssaviruses in bats [60], but was never proven to happen under natural conditions [61].

Aside from differences in pathogenicity, significantly longer incubation periods were observed following i.m. inoculation with two particular isolates when high doses were used (Fig 1b). Interestingly, both isolates have particular genetic characteristics, i.e. insertion in the NP-region. The longer incubation period of isolate 5006_EBLV-1b_ins also correlated with slow replication and low titres in one step replication kinetics. Whether this is due to the AA exchange S176P in the phospoprotein (S3 and S4 Tables) which might have an influence on interferon antagonism [62] or the observed six nucleotide insertion (AAAAGA) in the N-gene UTR is debatable (25). It needs to be clarified whether the insertion in front of the transcription termination signal (TTS) has an influence on termination and downstream transcription. In any case, this insertion seems to have no disadvantage for virus transmission and host maintenance under natural conditions as this insertion has been found in several subsequent EBLV-1b isolates from France, Germany [25, 26] and the Netherlands (B.Kooi, pers. Communication). Generally, 7As in the TTS are considered optimal for termination of transcription [63]. The single A insertion in Isolate 5776_EBLV-1a-ins leading to an 8A TTS affects transcription termination at the N and P gene border.

The incubation periods between the different inoculation routes and doses varied for the same isolates. In several cases the incubation periods following i.m. inoculation with high doses were even shorter than following i.c. inoculation. This is interesting since after i.m. inoculation the virus needs to travel from hind limb to the central nervous system in order to reach its main replication site. An explanation may be the dose of infection, whereby a thousand-fold higher dose was used for i.m. compared to i.c. inoculation. This is corroborated by the fact that incubation periods for 5989_RABV_dog_azerb and 5782_EBLV-1a_del following i.m. inoculation with low doses were significantly longer compared to i.c. inoculation (p-values: 0.0021 and 0.0023, resp., Fig 2a, 2b and 2c).

Clinical signs depended on the inoculation route as well as on the virus species. As for the latter, mice inoculated i.m. with the RABV isolates showed signs like ruffled fur, hunched back, slowed movements, tremor and paralysis whereas EBLV-1 infected mice displayed agitation and spasms. A difference between RABV and EBLV-1 had been described before [28], although here some RABV infected mice also exhibited sings of furious rabies.

Mice inoculated i.c. usually developed general clinical signs, whereas mice inoculated i.m. showed spasms and mice inoculated i.n. displayed aggressive behavior and circular movement. This is likely a result of the specific virus distribution in peripheral neurons and brain following the different routes of infection. Indeed, IHC analyses revealed that after i.n. inoculation antigen was more prevalent in the olfactory bulb and the overall brain, compared to i.m. inoculated mice. As for the olfactory bulb, it is comprehensible that there is more antigen present following i.n. compared to i.m. inoculation as virus has to travel through this brain section in order to infect the other parts of the brain. However, this does not explain the similar amount of antigen present in the medulla for both inoculation routes. Perhaps, clinical signs appear at a later stage of infection after i.n. infection when the virus is already present in most parts of the brain compared to i.m. infection. This would also explain the overall higher antigen content following i.n. infection compared to i.m. infection.

Following i.n. infection the fact that none of the mice seroconverted is not surprising since it was shown for RABV that sensory neurons in the olfactory mucosa are infected and the virus travels directly to the olfactory bulb [64], obviously without measurable interaction with the immune system. In contrast, a larger percentage of mice seroconverted following i.m. inoculation with high doses compared to low dose inoculation. Generally, there was no correlation between the serological response and the outcome of infection. In survivors that did not seroconvert, it is likely that the innate immune response was able to clear the virus without triggering an adaptive immune response (S3 Fig).

Full genome sequences were generated for all viruses used in this study and thus the number of available sequences more than doubled. Sequence analyses confirmed previously discovered unique indels in the selected EBLV-1 isolates. Additionally, NGS provided evidence for the presence of another single nt insertion in the G-UTR of isolate 5006_EBLV-1b_ins. The reported high sequence identity within EBLV-1 of >96.7% based on partial N-gene sequences [23, 24] was confirmed on full genome sequence level with the isolates from this study having an identity of 95.2% or higher.

On protein level, only two aa exchanges were found in known pathogenicity determining sites (S3 and S4 Tables). Their potential effect and the effect of the other 69 observed aa exchanges particularly those that resulted in a change of the respective aa property on the results of this study is difficult to assess. Against the background that most pathogenicity markers were determined using attenuated RABV strains, this needs further investigation.

The glycosylation site at position 319 in the glycoprotein was found in all isolates used in this study and is known to be conserved in at least seven lyssavirus species [30]. One additional glycosylation site was found at position 37 in the RABV isolates and a third glycosylation site is present in the isolate 35009_RABV_CVS at position 204. Wildtype RABV strains have usually two glycosylation sites but can acquire more during cell culture passage resulting in up to four glycosylation sites in certain fixed RABV strains [65]. Fixed RABV strains have certain advantages compared to wildtype viruses, e.g. fixed clinical picture, incubation periods and mortality rates [66]. Unfortunately, fixed virus strains are often attenuated following peripheral inoculation compared to wildtype viruses likely due to cell culture or host adaptation [67, 68]. This may explain why the fixed RABV strain 35009_RABV_CVS was highly attenuated compared to a wildtype isolate 5989_RABV_dog_azerb in this study (Fig 2b and 2c).

In order to generate virus for inoculation, all viruses used in our study had to be passaged in cell culture offering the possibility for adaption. However, none of the sequences derived from passaged material indicated nucleotide exchanges compared to previously generated partial sequences of the primary isolate. Furthermore, indels have not been described as result of cell-culture adaptation of lyssaviruses. Functionally, isolate 13027_EBLV-1_Yuli, which had the longest passage history of 11 passages on MNA cells, was still highly pathogenic following peripheral inoculation (Fig 2a).

### Conclusion

Although EBLV-1 isolates display very high sequence conservation, significant differences in pathogenicity as well as in incubation periods were found. Thus, transfer of results obtained with single isolates to the whole lyssavirus species can be misleading. The cause of these differences can only be speculated, as data concerning pathogenicity determinants, especially for EBLV-1, are insufficient. Here further studies using reverse genetics are warranted to confirm the role of indels as well as SNPs. To this end, isolate 13454_EBLV-1a_ref already available as recombinant virus [37], was included in the study. Retrospectively, results indicating reduced pathogenicity obtained with EBLV-1 isolates in previous studies have to be carefully interpreted. Thus, the results emphasize the need for proper post-exposure prophylaxis in case of any severe exposure to the reservoir hosts of lyssaviruses.

## Supporting information

S1 Fig a) two step and b) one step replication kinetics of the isolates used in the study.
(PDF)

S2 Fig Survival curves of the mice following i.c. inoculation. (PDF)

S3 Fig Percentage seroconversion for the different inoculation routes following inoculation a) with EBLV-1 isolates and b) with RABV isolates. Percentage of seroconverted mice for the individual isolates can be seen following i.m. inoculation with c) high doses and d) low doses.
(PDF)

S1 Table Clinical score sheet of the mice, ranging from zero up to four. (PDF)

S2 Table The 28 potentially significant amino acid exchanges in the proteins of the EBLV-1 isolates and their frequency in other EBLV-1 as well as in RABV isolates. (PDF)

S3 Table Summary of known pathogenicity determining sites and the protein sequences found in the RABV as well as in the EBLV-1 isolates used in this study. (PDF)

S4 Table Summary of previous pathogenicity studies with EBLV-1 as well as details to their experimental design. 13454* is identical to 13454_EBLV-1a_ref used in this study.
(PDF)
