# Supplementary figures and images for: Comparative analysis of European bat lyssavirus 1 pathogenicity in the mouse model
Source: PLoS Negl Trop Dis. 2017 Jun 19;11(6):e0005668. doi: 10.1371/journal.pntd.0005668 (PMC5491315; doi:10.1371/journal.pntd.0005668)

a)

MOI: 0.01

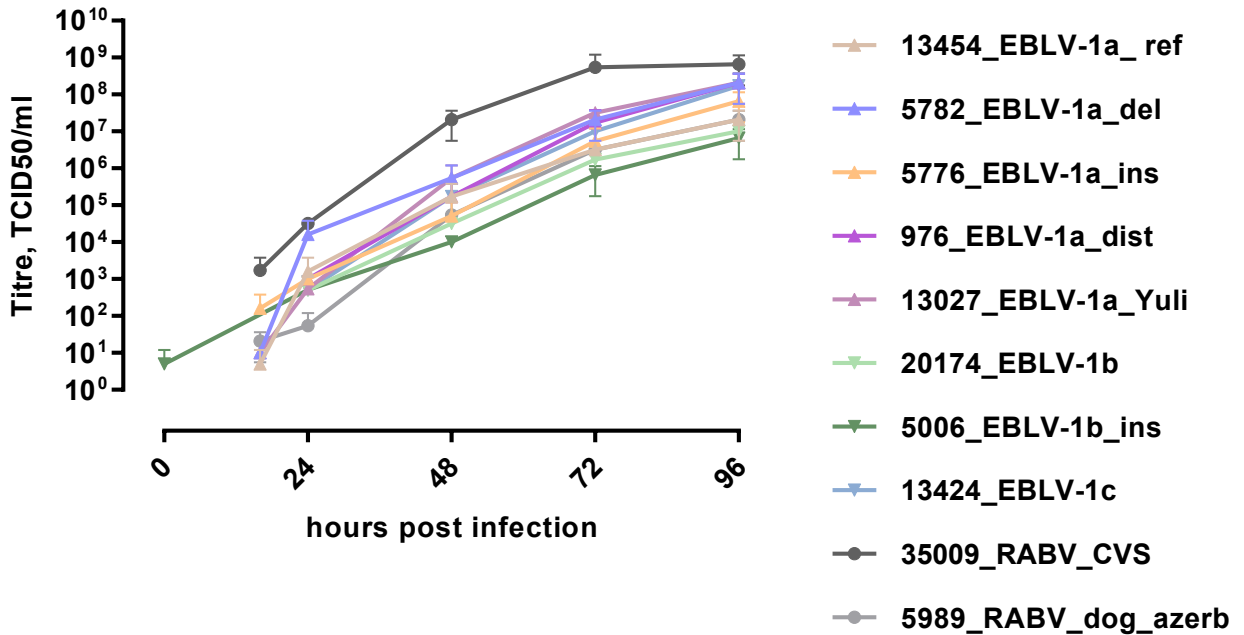

b)

MOI: 3

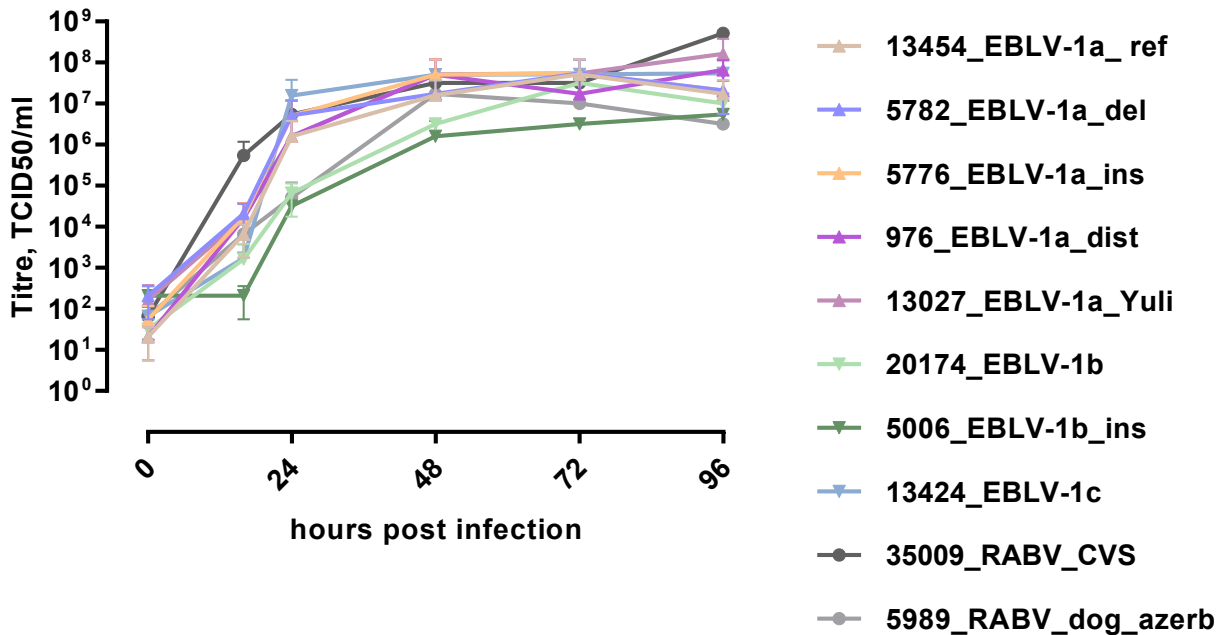

Supplement: S1 Fig — a) two step and b) one step replication kinetics of the isolates used in the study. (PDF) [file pntd.0005668.s001.pdf]

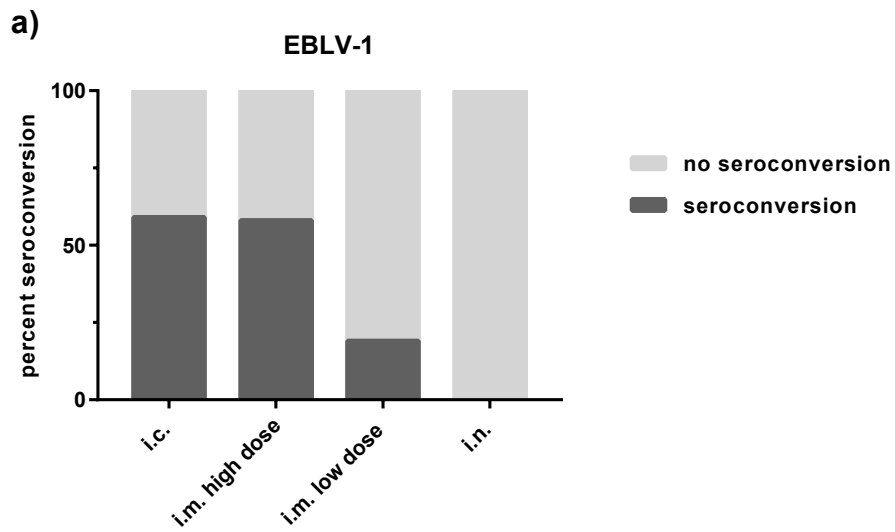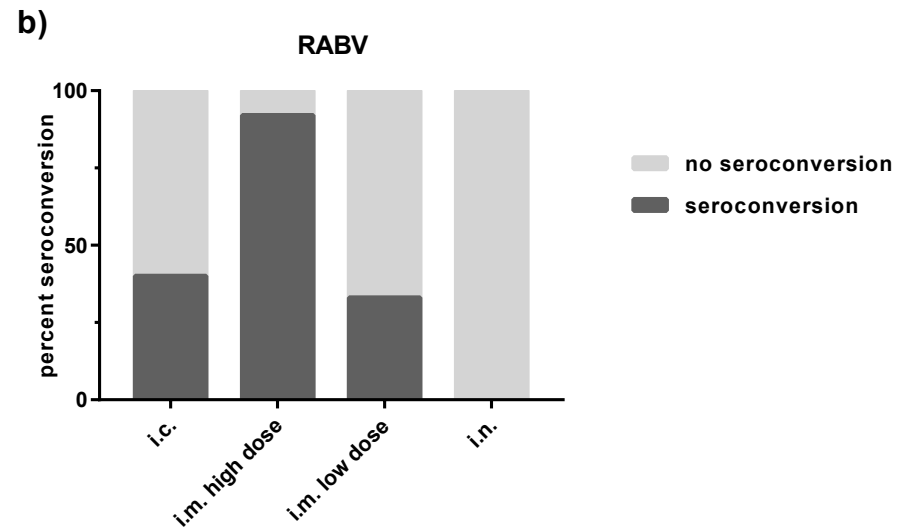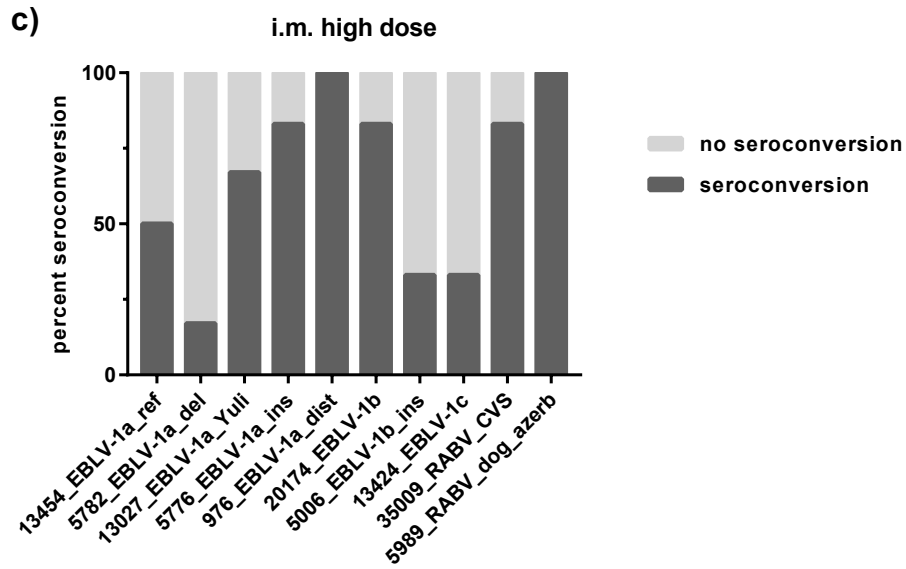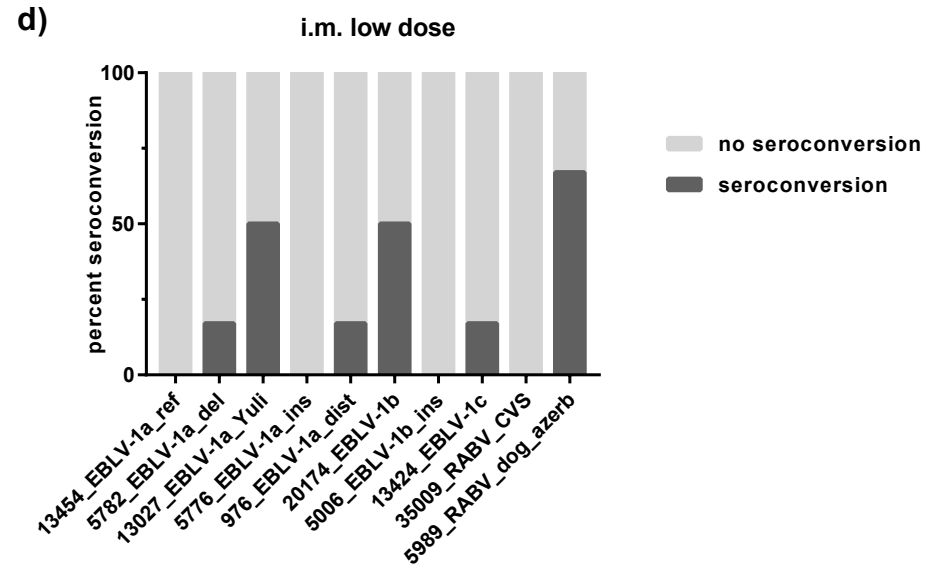

Supplement: S3 Fig — Percentage seroconversion for the different inoculation routes following inoculation a) with EBLV-1 isolates and b) with RABV isolates. Percentage of seroconverted mice for the individual isolates can be seen following i.m. inoculation with c) high doses and d) low doses. (PDF) [file pntd.0005668.s003.pdf]
