# Supplementary material for: Comparative analysis of European bat lyssavirus 1 pathogenicity in the mouse model
Source: PLoS Negl Trop Dis. 2017 Jun 19;11(6):e0005668. doi: 10.1371/journal.pntd.0005668 (PMC5491315; doi:10.1371/journal.pntd.0005668)
Supplement: S2 Fig — (PDF) [file pntd.0005668.s002.pdf]

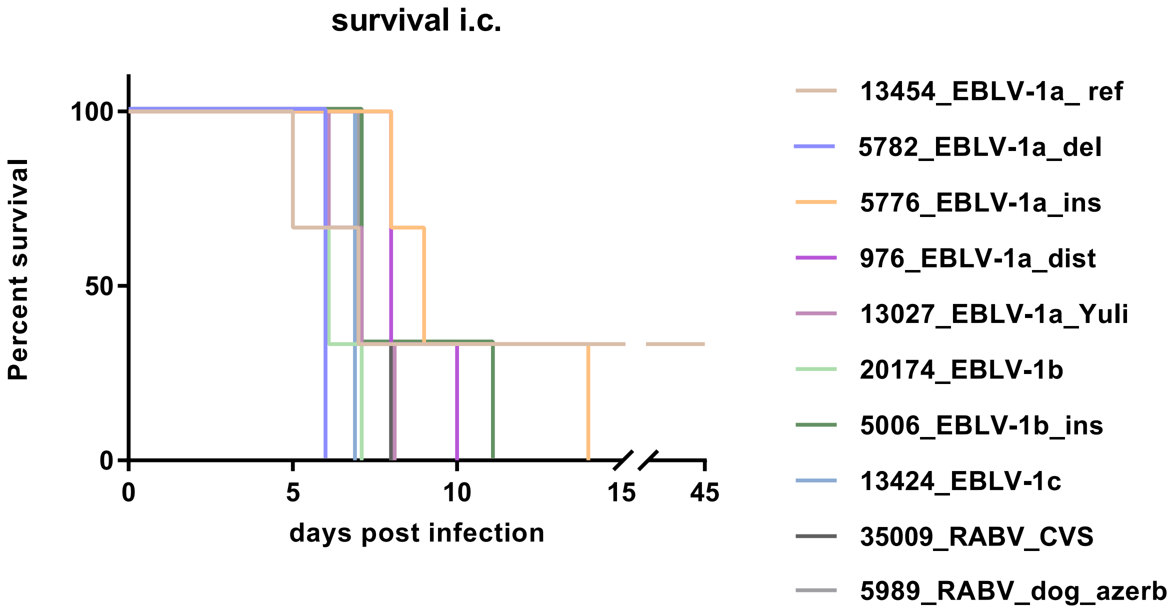

survival i.c.
Percent survival
100
50
0
0
5
10
15
45
days post infection
13454_EBLV-1a_ ref
5782_EBLV-1a_del
5776_EBLV-1a_ins
976_EBLV-1a_dist
13027_EBLV-1a_Yuli
20174_EBLV-1b
5006_EBLV-1b_ins
13424_EBLV-1c
35009_RABV_CVS
5989_RABV_dog_azerb
