# Supplementary material for: Comparative analysis of European bat lyssavirus 1 pathogenicity in the mouse model
Source: PLoS Negl Trop Dis. 2017 Jun 19;11(6):e0005668. doi: 10.1371/journal.pntd.0005668 (PMC5491315; doi:10.1371/journal.pntd.0005668)
Supplement: S1 Table — (PDF) [file pntd.0005668.s004.pdf]

Score	Symptoms
0	• healthy
1	• ruffled fur<br>• hunched back<br>• hypermetria in inoculated limb<br>• wobbly gait in inoculated limb<br>• calm
2	• ruffled fur<br>• hunched back<br>• slow movements<br>• wobbly gait both hind limbs<br>• jumpy<br>• tame
3	• paralysis or spasms in hind limbs<br>• agressiveness<br>• biting of objects and other mice<br>• automutilation<br>• circular movements
4	• death
