# Supplementary material for: Comparative analysis of European bat lyssavirus 1 pathogenicity in the mouse model
Source: PLoS Negl Trop Dis. 2017 Jun 19;11(6):e0005668. doi: 10.1371/journal.pntd.0005668 (PMC5491315; doi:10.1371/journal.pntd.0005668)
Supplement: S2 Table — (PDF) [file pntd.0005668.s005.pdf]

Gene	N-gene		P-gene						M-gene						G-gene								L-gene					
Position	90	112	61	140	141	156	157	176	29	56	85	121	140	163	-1	23	80	167	256	336	496	501	132	860	1075	1786	2097	2101
13454_EBLV-1a _ref	T	H	Y	L	Q	T	A	S	D	H	E	I	Q	H	A	S	T	I	Q	K	S	A	K	Y	H	A	Q	K
5782_EBLV-1a_del	.	.	.	.	.	A	.	.	.	N	G	.	.	.	.	R	.	.	.	.	L	.	.	.	.	.	.	.
5776_EBLV-1a_ins	.	.	.	.	.	A	.	.	.	N	.	.	.	.	.	.	.	.	.	.	.	.	.	.	.	.	.	.
976_EBLV-1a _dist	.	.	.	.	.	A	.	.	.	N	G	.	K	.	.	.	I	.	.	.	.	.	.	.	.	.	.	.
13027_EBLV-1a_Yuli	.	.	.	.	.	A	.	.	Y	N	G	R	.	.	.	.	.	.	.	.	.	.	N	.	.	.	.	.
20174_EBLV-1b	.	N	.	P	H	A	T	.	.	N	G	.	.	.	G	.	.	.	.	T	.	T	.	F	.	T	K	.
5006_EBLV-1b_ins	.	N	C	P	H	A	T	P	.	N	G	.	.	.	G	.	.	T	.	T	.	T	.	F	.	T	K	Q
13424_EBLV-1c	I	N	.	P	H	A	T	.	.	N	G	.	.	P	G	T	.	.	R	T	.	T	.	F	N	T	K	.
Other EBLV-1 isolates	I: 0%	N:45.1%; S:1.2%	C: 0%	P: 28.6%	H: 28.6%	T: 42.9%	T: 28.6%	P: 0%	Y: 0%	H: 0%	E: 0%	R: 0%	K: 0%	P: 0%	G: 39.3%	R: 5.4%, T:0%	I: 0%	T: 3.6%	R: 0%	T: 39.3%	L: 0%	T: 39.3%, V: 1.8%	N: 0%	F: 28.6%	N: 0%	T: 28.6%	K: 28.6%	Q: 14.3%
Number of AA sequences (EBLV-1)	82 sequences		7 sequences						9 sequences						56 sequences								7 sequences					
RABV-isolates	T: 91.8%; N:3.9%; I:2.8%; V:1.3%; A:0.2%	K:82.9; R:16.4; M:0.4; N&Q: 0.1%	G: 50.6%; E: 21.3%; K: 17.2%; R: 4.7%; D: 4.5%; T: 1.2%; A: 0.2%	S: 78%; P: 20.1%; V: 1.2%; F: 0.5%; A: 0.2%	S: 80.1%; L: 18.5%; T: 1.2%; V: 0.2%	E: 97.7%; D: 0.5%; K: 0.2%;	T: 90.6%; A: 5.0%; P: 1.6%; V,S: 0.5%; M: 0.2%	A: 99.1%; V: 0.5%; T: 0.3%	D: 100%	N: 99.6% K, S: 0.2%	G: 96.9%; W: 3.1%	I: 99.0%; V: 1.0%	Q: 100%	H: 100%	G: 99.9%; W: 0.1%	S: 99.4%; R: 0.2%; N,G: 0.1%	T: 100%	T: 99.2%; A: 0.6%; I: 0.1%	Q: 96.8%; R; 2.8%; K: 0.4%	N: 93.2%; D: 3.8%; S: 2.9%; G: 0.1%	S: 82.9%; L: 14.4%; Y: 2.4%; V: 0.1%	G: 85.7%; S: 13.1%; A,D: 0.4%; N: 0.2%; C: 0.1%	K: 85.1%; R: 14.3%; N: 0.6%	F: 99.4%; Y: 0.3%; X: 0.3%	H: 99.4%; X: 0.6%	Q: 85.7%; K: 11.0%; R: 3.0%; H: 0.3%	G: 73.5%; R: 20.4%; K: 6.1%	R: 60.4%; K: 39.6%
Number of AA sequences (RABV)	2557 sequences		576 sequences						520 sequences						2264 sequences								327 sequences (RABV-Alignment +1AA)					
