# Supplementary material for: Comparative analysis of European bat lyssavirus 1 pathogenicity in the mouse model
Source: PLoS Negl Trop Dis. 2017 Jun 19;11(6):e0005668. doi: 10.1371/journal.pntd.0005668 (PMC5491315; doi:10.1371/journal.pntd.0005668)
Supplement: S3 Table — (PDF) [file pntd.0005668.s006.pdf]

	Nucleoprotein		Phosphoprotein			Matrixprotein				Glycoprotein									Large protein			
Reference	(Masatani, Ito et al. 2011)	(Kgaladi, Wright et al. 2013)	(Kgaladi, Wright et al. 2013)	(Rieder, Brzozka et al. 2011)	(Brzozka, Finke et al. 2006)	(Wirblich, Tan et al. 2008)	(Kgaladi, Wright et al. 2013)	(Gholami, Kassis et al. 2008)	(Mita, Shimizu et al. 2008)	(Faber, Faber et al. 2005)	(Lentz, Wilson et al. 1984)	(Takayama-Ito, Ito et al. 2006)	(Kgaladi, Wright et al. 2013)	(Kgaladi, Wright et al. 2013)	(Langevin and Tuffereau 2002)	(Kgaladi, Wright et al. 2013)	(Tuffereau, Leblois et al. 1989)	(Prehaud, Wolff et al. 2010)	(Tian, Luo et al. 2015)			
position	AA273	AA394	AA 144-148	AA176-186	AA289-298	AA35-38	AA77	AA81	AA95	AA 194	AA198	AA242	AA255	AA268	AA318	AA352	AA330-333	AA502-506	AA1685	AA1797	AA1829	AA1867
13454_EBLV-1a_ref	F	F	KSTQT	SQESSGPPGLD	QDDLNRYLAY	PPEY	S	G	V	T	K	S	D	I	I	H	KSVR	TGES	K	D	K	E
5782_EBLV-1a_del	F	F	KSTQT	SQESSGPPGLD	QDDLNRYLAY	PPEY	S	G	V	T	K	S	D	I	I	H	KSVR	TGES	K	D	K	E
5776_EBLV-1a_ins	F	F	KSTQT	SQESSGPPGLD	QDDLNRYLAY	PPEY	S	G	V	T	K	S	D	I	I	H	KSVR	TGES	K	D	K	E
976_EBLV-1a_dist	F	F	KSTQT	SQESSGPPGLD	QDDLNRYLAY	PPEY	S	G	V	T	K	S	D	I	I	H	KSVR	TGES	K	D	K	E
13027_EBLV-1a_Yuli	F	F	KSTQT	SQESSGPPGLD	QDDLNRYLAY	PPEY	S	G	V	T	K	S	D	I	I	H	KSVR	TGES	K	D	K	E
20174_EBLV-1b	F	F	KSTQT	SQESSGPPGLD	QDDLNRYLAY	PPEY	S	G	V	T	K	S	D	I	I	H	KSVR	TSES	K	D	K	E
5006_EBLV-1b_ins	F	F	KSTQT	PQESSGPPGLD	QDDLNRYLAY	PPEY	S	G	V	T	K	S	D	I	I	H	KSVR	TSES	K	D	K	E
13424_EBLV-1c	F	F	KSTQT	SQESSGPPGLD	QDDLNRYLAY	PPEY	S	G	V	T	K	S	D	I	I	H	KSVR	TSES	K	D	K	E
35009_RABV_CVS	F	Y	KSTQT	AQVAPGPPALE	QDDLNRYTSC	PPEY	R	E	V	N	K	A	D	I	F	H	KSVR	EIRL	K	D	K	E
5989_RABV_dog_azerb	F	Y	KSTQT	AQAASGPPALE	QDDLNRYASC	PPEY	R	E	V	N	K	A	D	I	F	H	KSVR	ETRL	K	D	K	E

References:

Brzozka, K., S. Finke and K. K. Conzelmann (2006). "Inhibition of interferon signaling by rabies virus phosphoprotein P: activation-dependent binding of STAT1 and STAT2." J Virol 80(6): 2675-2683.

Faber, M., M. L. Faber, A. Papaneri, M. Bette, E. Weihe, B. Dietzschold and M. J. Schnell (2005). "A Single Amino Acid Change in Rabies Virus Glycoprotein Increases Virus Spread and Enhances Virus Pathogenicity." Journal of Virology 79(22): 14141-14148.

Gholami, A., R. Kassis, E. Real, O. Delmas, S. Guadagnini, F. Larrous, D. Obach, M. C. Prevost, Y. Jacob and H. Bourhy (2008). "Mitochondrial dysfunction in lyssavirus-induced apoptosis." Journal of Virology 82(10): 4774-4784.

Kgaladi, J., N. Wright, J. Coertse, W. Markotter, D. Marston, A. R. Fooks, C. M. Freuling, T. F. Müller, C. T. Sabeta and L. H. Nel (2013). "Diversity and Epidemiology of Mokola Virus." PLoS Negl Trop Dis 7(10): e2511.

Langevin, C. and C. Tuffereau (2002). "Mutations conferring resistance to neutralization by a soluble form of the neurotrophin receptor (p75NTR) map outside of the known antigenic sites of the rabies virus glycoprotein." J Virol 76(21): 10756-10765.

Lentz, T. L., P. T. Wilson, E. Hawrot and D. W. Speicher (1984). "Amino acid sequence similarity between rabies virus glycoprotein and snake venom curaremimetic neurotoxins." Science 226(4676): 847-848.

Masatani, T., N. Ito, K. Shimizu, Y. Ito, K. Nakagawa, M. Abe, S. Yamaoka and M. Sugiyama (2011). "Amino acids at positions 273 and 394 in rabies virus nucleoprotein are important for both evasion of host RIG-I-mediated antiviral response and pathogenicity." Virus Res 155(1): 168-174.

Mita, T., K. Shimizu, N. Ito, K. Yamada, Y. Ito, M. Sugiyama and N. Minamoto (2008). "Amino acid at position 95 of the matrix protein is a cytopathic determinant of rabies virus." Virus Research 137(1): 33-39.

Prehaud, C., N. Wolff, E. Terrien, M. Lafage, F. Megret, N. Babault, F. Cordier, G. S. Tan, E. Maitrepierre, P. Menager, D. Chopy, S. Hoos, P. England, M. Delepierre, M. J. Schnell, H. Buc and M. Lafon (2010). "Attenuation of rabies virulence: takeover by the cytoplasmic domain of its envelope protein." Sci Signal 3(105): ra5.

Rieder, M., K. Brzozka, C. K. Pfaller, J. H. Cox, L. Stitz and K. K. Conzelmann (2011). "Genetic dissection of interferon-antagonistic functions of rabies virus phosphoprotein: inhibition of interferon regulatory factor 3 activation is important for pathogenicity." J Virol 85(2): 842-852.

Takayama-Ito, M., N. Ito, K. Yamada, M. Sugiyama and N. Minamoto (2006). "Multiple amino acids in the glycoprotein of rabies virus are responsible for pathogenicity in adult mice." Virus Res. 115(2): 169-175.

Tian, D., Z. Luo, M. Zhou, M. Li, L. Yu, C. Wang, J. Yuan, F. Li, B. Tian, B. Sui, H. Chen, Z. F. Fu and L. Zhao (2015). "Critical Role of K1685 and K1829 in the Large Protein of Rabies Virus in Viral Pathogenicity and Immune Evasion." J Virol 90(1): 232-244.

Tuffereau, C., H. Leblois, J. Benejean, P. Coulon, F. Lafay and A. Flamand (1989). "Arginine or lysine in position 333 of ERA and CVS glycoprotein is necessary for rabies virulence in adult mice." Virology 172(1): 206-212.

Wirblich, C., G. S. Tan, A. Papaneri, P. J. Godlewski, J. M. Orenstein, R. N. Harty and M. J. Schnell (2008). "PPEY motif within the rabies virus (RV) matrix protein is essential for efficient virion release and RV pathogenicity." J Virol **82**(19): 9730-9738.
