# Supplementary material for: Comparative analysis of European bat lyssavirus 1 pathogenicity in the mouse model
Source: PLoS Negl Trop Dis. 2017 Jun 19;11(6):e0005668. doi: 10.1371/journal.pntd.0005668 (PMC5491315; doi:10.1371/journal.pntd.0005668)
Supplement: S4 Table — 13454* is identical to 13454_EBLV-1a_ref used in this study. (PDF) [file pntd.0005668.s007.pdf]

Isolate	cells for virus propagation	Mice	Age of mice	Number per group	Inoculation dose	Inoculation route	Incubation time (days)	Mortality	Other	Reference
13454*	MNA	CD1-mice	-	5	10^4,5 FFU	i.m., M. glutaeus	8-9	100%	-	(Vos, Müller et al. 2004)
13454*	MNA	CD1-mice	-	5	10^2,5 FFU	i.m., M. glutaeus	11-15	60%	-	(Vos, Müller et al. 2004)
Stade isolate	BSR	OF1 Swiss mice	4 weeks	8-9	10^7 PFU	i.m., hind leg	7-14	>75%	-	(Montano-Hirose, Lafage et al. 1993)
RV20 (bat denmark)	BHK	RIII inbred albino	3-4 weeks	5	25 MLD50	i.c./ f.p.	-	100%/ 40%	10^6 TCID50 (i.c. 10^4.5 MLD50, f.p. 10^2.9 MLD50)	(Brookes, Parsons et al. 2005)
RV20 (bat denmark)	BHK	RIII inbred albino	3-4 weeks	5	2.5 MLD50	i.c./ f.p.	-	100%/ 20%	10^6 TCID50 (i.c. 10^4.5 MLD50, f.p. 10^2.9 MLD50)	(Brookes, Parsons et al. 2005)
RV20 (bat denmark)	BHK	RIII inbred albino	3-4 weeks	5	0.25 MLD50	i.c./ f.p.	-	60%/ 0%	10^6 TCID50 (i.c. 10^4.5 MLD50, f.p. 10^2.9 MLD50)	(Brookes, Parsons et al. 2005)
RV1423	-	CD1 mice	5 weeks	25	8*10^2 infectious virus particles	f.p.	-	32%	-	(Hicks, Nunez et al. 2013)
RV1423	-	OF1 Swiss mice	5 weeks	7	5 MLD50	f.p.	8-11	100%	-	(Healy, Brookes et al. 2013)
RV1423	-	OF1 Swiss mice	6 weeks	13	5 MLD50	f.p.	7-13	100%	-	(Hicks, Nunez et al. 2009)
EBL1Fra	-	BALB/c and C3H	6-8 weeks	8	10^5 MICLD50	i.m. tigh	13-17	100%	-	(Badrane, Bahloul et al. 2001)
-	Neuro-2a, BHK21-C13	BALB/c	7-8 weeks	-	3x10^7 MICLD50	i.m. hind leg	9-13 (surv. time)	100%	-	(Perrin, DeFranco et al. 1996)

FFU: Foci forming units, PFU: Plaque forming units; MLD50: Mouse lethal dose 50; MICLD50: Mouse intracerebral lethal dose 50; i.m.: intramuscular; f.p.: footpad;

References:

Vos, A., T. Müller, J. Cox, L. Neubert and A. R. Fooks (2004). "Susceptibility of ferrets (Mustela putorius furo) to experimentally induced rabies with European Bat Lyssaviruses (EBLV)." Journal of Veterinary Medicine Series B **51**(2): 55-60.

Montano-Hirose, J. A., M. Lafage, P. Weber, H. Badrane, N. Tordo and M. Lafon (1993). "Protective activity of a murine monoclonal antibody against European bat lyssavirus 1 (EBL1) infection in mice." Vaccine **11**(12): 1259-1266.

Brookes, S. M., G. Parsons, N. Johnson, L. M. McElhinney and A. R. Fooks (2005). "Rabies human diploid cell vaccine elicits cross-neutralising and cross-protecting immune responses against European and Australian bat lyssaviruses." Vaccine **23**(32): 4101-4109.

Hicks, D. J., A. Nunez, A. C. Banyard, A. Williams, A. Ortiz-Pelaez, A. R. Fooks and N. Johnson (2013). "Differential chemokine responses in the murine brain following lyssavirus infection." J Comp Pathol **149**(4): 446-462.

Healy, D. M., S. M. Brookes, A. C. Banyard, A. Núñez, S. L. Cosby and A. R. Fooks (2013). "Pathobiology of rabies virus and the European bat lyssaviruses in experimentally infected mice." Virus Research **172**(1–2): 46-53.

Hicks, D. J., A. Nunez, D. M. Healy, S. M. Brookes, N. Johnson and A. R. Fooks (2009). "Comparative pathological study of the murine brain after experimental infection with classical rabies virus and European bat lyssaviruses." J Comp Pathol **140**(2-3): 113-126.

Badrane, H., C. Bahloul, P. Perrin and N. Tordo (2001). "Evidence of two Lyssavirus phylogroups with distinct pathogenicity and immunogenicity." J Virol **75**(7): 3268-3276.

Perrin, P., M. T. DeFranco, C. Jallet, F. Fouque, S. Morgeaux, N. Tordo and J. H. Colle (1996). "The antigen-specific cell-mediated immune response in mice is suppressed by infection with pathogenic lyssaviruses." Research in Virology **147**(5): 289-299.
